# Supplementary material for: Diagnosis and Antibiotic Treatment of Urinary Tract Infections in Danish General Practice: A Quality Assessment
Source: Antibiotics (Basel). 2022 Dec 5;11(12):1759. doi: 10.3390/antibiotics11121759 (PMC9774586; doi:10.3390/antibiotics11121759)
Supplement: Supplementary file 1 [file antibiotics-11-01759-s001.zip › antibiotics-2064341-supplementary.pdf]

Supplementary Material

Suspected urinary tract infections in patients ≥ 18 years

Register solely on the basis of the day of the first contact to general practice (day one)

**Patient information**		
1	Patient age	______ years
2	Patient sex	☐ Female<br>☐ Male
3	Patient background<br>(choose at least 1)	☐ Nursing home resident<br>☐ Urinary catheter<br>☐ Recurring UTI<br>☐ Comorbidity<br>☐ Pregnant<br>☐ Penicillin allergy<br><br>☐ None of the above
**Symptoms and signs**		
4	Symptoms and signs<br>(choose at least 1)	☐ Dysuria<br>☐ Urgency<br>☐ Frequency<br>☐ New-onset incontinence<br>☐ Suprapubic pain<br>☐ Flank pain<br>☐ Fever<br>☐ Shivering<br>☐ Systemically unwell<br>☐ New-onset confusion<br>☐ None of the above<br><br>☐ No symptoms reported
**Examinations**		
5	Urinary dipstick<br>(choose at least 1)	**Nitrite**<br>☐ Positive<br>☐ Negative<br>**Leukocytes**<br>☐ Positive<br>☐ Negative<br>**Hemoglobin**<br>☐ Positive<br>☐ Negative<br><br>☐ Not performed

**Examinations** (continued)		
6	Microscopy<br>(choose only 1)	**Microscopy**<br>☐ Positive<br>☐ Negative<br>☐ Inconclusive<br><br>☐ Not performed
7	Urine culture<br>(choose only 1)	☐ Urine culture in general practice<br>☐ Susceptibility testing in general practice<br>☐ Urine sent to Department of Clinical Microbiology<br><br>☐ Not performed
8	CRP<br>(mg/l or 1 cross)	CRP (value in mg/L) _________<br><br>☐ Not performed
**Diagnose**		
9	Diagnose<br>(choose only 1)	☐ Uncomplicated lower UTI<br>☐ Complicated lower UTI<br>☐ Pyelonephritis<br>☐ Other, not UTI<br>☐ Unresolved
**Action**		
10	Hospitalisation<br>(choose only 1)	☐ Yes<br>☐ No
11	Antibiotics<br>(choose at least 1)	☐ Pivmecillinam<br>☐ Sulfametizol<br>☐ Nitrofurantoin<br>☐ Trimethoprim<br>☐ Ciprofloxacin<br>☐ Other antibiotic<br><br>☐ No antibiotics (day 1)
